# Nanoaerosols reduce required effective dose of liposomal levofloxacin against pulmonary murine *Francisella tularensis* subsp. *novicida* infection

**DOI:** 10.1186/s12951-016-0182-0

**Published:** 2016-04-18

**Authors:** Crystal N. Propst, Albert O. Nwabueze, Igor L. Kanev, Rachel E. Pepin, Bradford W. Gutting, Victor N. Morozov, Monique L. van Hoek

**Affiliations:** School of Systems Biology and National Center for Biodefense and Infectious Diseases, George Mason University, MS1H8, Manassas, VA 20110 USA; CBR Concepts and Experimentation Branch (Z21), Dahlgren Division, Naval Surface Warfare Center, Dahlgren, VA 22448 USA; Institute of Theoretical and Experimental Biophysics of the Russian Academy of Sciences, Pushchino, Moscow Region, 142290 Russia

**Keywords:** *Francisella*, Aerosol, Pulmonary infection, Levofloxacin, Animal model, Nanoaerosol

## Abstract

**Background:**

The Institute of Theoretical and Experimental Biophysics in Moscow recently developed a new nanoaerosol generator. This study evaluated this novel technology, which has the potential to enhance therapeutic delivery, with the goal of using the generator to treat pulmonary *Francisella tularensis* subsp. *novicida* (*F. novicida*) infections in BALB/c mice.

**Results:**

First, the analysis of quantum dots distribution in cryosections of murine lungs demonstrated that nanoaerosols penetrate the alveoli and spread more homogenously in the lungs than upon intranasal delivery. Second, the generator was used to aerosolize the antibiotic levofloxacin to determine the effectiveness of nanoaerosolized levofloxacin as treatment against *F. novicida*. The generator was capable of delivering a sufficient dose of nanoaerosolized liposome-encapsulated levofloxacin to rescue mice against 100LD_50_ of *F. novicida*.

**Conclusions:**

The nanoaerosol-delivered dosage of liposome-encapsulated levofloxacin required to rescue mice is approximately 94× lower than the oral required dose and approximately 8× lower than the intraperitoneal dose required for rescue. In addition, treatment with nanoaerosols consumes less total volume of therapeutic solutions and is gentler on sprayed material than the aerosolization by a conventional three-jet Collison nebulizer as seen by the preservation of liposomes. This could represent a significant advance for the use of expensive therapeutics and lung directed therapies.

**Electronic supplementary material:**

The online version of this article (doi:10.1186/s12951-016-0182-0) contains supplementary material, which is available to authorized users.

## Background

Aerosolized therapeutics improve upon traditional delivery methods in cases of pulmonary infection due to their ease of administration, access to the large lung surface area, and limited systemic distribution [1]. It has been found that the delivery of therapeutics via the pulmonary route bypasses digestive destruction in the stomach and first-pass metabolism in the liver, thus potentially greatly reducing required doses and resulting side effects. The merits of inhalational therapies have been well established in the treatment of cystic fibrosis patients where the high local concentration but low systemic effects are ideal [2].

Nanoaerosols are defined here as being aerosols comprised of particles that are less than 200 nm in diameter [3]. In comparison, aerosol particles created by a standard 3-jet Collison nebulizer are usually between 1 and 5 μm in diameter [4]. New technology in the production of nanoaerosols may allow for further improvement on the treatment of lung-based infections due to the enhanced deposition of therapeutic aerosol in the lower respiratory tract and appropriate localization in the alveoli, which may result in a lower necessary dose.

The Institute of Theoretical and Experimental Biophysics (ITEB) in Moscow has developed a new technology for the generation of nanoaerosolized biological materials, which can retain structural and functional properties of the molecules [5, 6]. This technology is based on the electrohydrodynamic atomization of a solution followed by gas-phase neutralization of the electrospray–generated ions and nanoclusters with oppositely charged ions generated via the same technique. It provides a new avenue in the fabrication of a variety of nanoproducts based on the fact that oppositely charged species are forced to form complexes upon neutralization, while collisions between similarly charged products are inhibited. Unlike the current methods of neutralization with corona, which generates highly reactive radicals, the electrospray-neutralization (ESN) technology employs less reactive ions and induces less damage to the sprayed material [7]. It has been demonstrated that ESN can produce protein nanoaerosols with almost complete retention of the functional properties of protein molecules, which speaks to its gentle aerosolization process and suitability for use with biologics and therapeutics [3, 8]. The size of the nanoaerosolized particles depends on a variety of parameters, the most important of which are drug concentration and flow rate. The full list of parameters and their effects are discussed in the references presented above.

Nanoaerosols generated by other methods have been shown to be an effective way to deliver anti-inflammatory and anti-hypertensive drugs to mice [9–11]. Due to these results, it seems logical and potentially beneficial to extend these studies to other therapeutics. In this respect, the new electrospray neutralization (ESN) aerosolization technology has an advantage over the harsh sublimation–condensation technique used in the anti-inflammatory and anti- hypertensive drug studies because it enables the generation of nanoaerosols from virtually any soluble substance. To demonstrate the ability to nanoaerosolize useful antibiotic therapeutics and compare treatment efficiency to traditional delivery methods, a mouse model of pulmonary *Francisella tularensis* subsp. *novicida* (*F. novicida*) infection was used.

The *Francisella* species are gram-negative, facultatively intracellular, pathogenic bacteria readily found in nature that can lead to a lethal infection in humans when as few as ten bacteria are inhaled [12]. Respiratory tularemia has been reported to result from traditional farming methods, lawn mowing, or otherwise aerosolizing contaminated materials [13]. However, the intentional exposure of humans to respiratory tularemia is a matter of great concern in the field of bioterrorism [13]. The United States’ Department of Health and Human Services has listed *F. tularensis* as a select agent due to its severe threat to both human and animal health, high degree of infectiousness, ability to be aerosolized, and the lack of a viable vaccine. In addition, *F. tularensis* has an additional notation as a Tier 1 Select Agent due to its history of being developed as a biological weapon by the United States and Soviet Union.

Levofloxacin is a third generation fluoroquinolone antibiotic shown to be highly effective in treating *Francisella* infections (MIC_90_ = 0.012 mg/L), despite not being considered the standard treatment [14–17]. Levofloxacin is well tolerated by most individuals, able to reach high blood levels and required MIC levels, capable of intracellular penetration, and has a lower relapse rate than standard treatments [16]. Due to the increasing number of cases of naturally acquired antibiotic resistance among pathogens, including *Francisella* species, and the possibility of the creation of genetically modified bacterial strains, it is critical that the scientific community investigate new or improved treatments against potential threat agents [18–20].

Wong et al. reported an increase in survival against 10LD_50_*F. tularensis* subsp. *LVS* by encapsulating ciprofloxacin in liposomes and delivering them via standard aerosol. The act of encapsulating ciprofloxacin in liposomes brought the survival from 0 % with a mean time to death of 8.2 days to 100 % [21]. Similarly, Hamblin et al. reported that aerosolized liposome-encapsulated ciprofloxacin was capable of rescuing mice from a lethal *F. tularensis* subsp. *Schu S4* infection with as little as a single aerosol treatment [22]. Levofloxacin encapsulated in liposomes could enhance treatments against tularemia compared to levofloxacin alone.

This study investigated the utility of a nanoaerosol-based therapeutic approach using levofloxacin against a murine pulmonary *Francisella* infection as a model. Furthermore, this therapeutic approach was compared in the same animal model to traditional delivery methods: intraperitoneal injection, oral administration, and 3-jet Collison nebulizer generated aerosols.

## Results and discussion

**Fig. 1:**
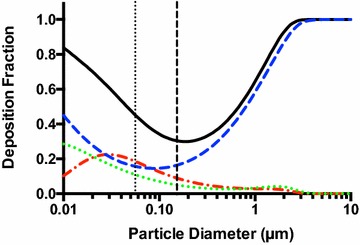
Predicted respiratory deposition of various sized particles. This MPPD 3.0 simulation shows deposition fraction of particles in the total (*black*, *solid*), nasal (*blue*, *dashed*) tracheobronchial (*green*, *dotted*), and alveolar (*red*, *alternating*) regions based on diameter. The *vertical dotted line* signifies mean diameter of nanoaerosolized levofloxacin and the *vertical dashed line* signifies mean diameter of nanoaerosolized liposome-encapsulated levofloxacin

MPPD 3.0 software models the deposition of various sized aerosolized particles within a mouse respiratory tract based on extensive data from previously published studies [23]. As seen in Fig. 1, large particle aerosols have the highest total deposition in the lungs but a very small percentage of that is deposited in the lower respiratory tract. Small particle aerosols have a lower total deposition but a large portion of these particles is retained in the alveoli. In an effort to maximally target the alveoli for the purpose of increasing treatment efficiency, nano-sized particles should be used.

Based on this data, it can be seen that particles generated by the 3-jet Collison nebulizer, which range from 1 to 5 μm [4], have a high total deposition but that those particles mostly deposit in the nasal cavity, as there is an extremely low percentage of alveolar and tracheobronchial deposition for particles of that size. Particles generated by the ESN generator fit the nano-size range that is predicted to have less total deposition but a significantly higher deposition percentage in the alveolar and tracheobronchial regions. It is hypothesized that the increased deposition of therapeutics in the lower respiratory tract through the use of the ESN generator will contribute to an improved outcome against pulmonary infections despite the low overall deposition.

**Fig. 2:**
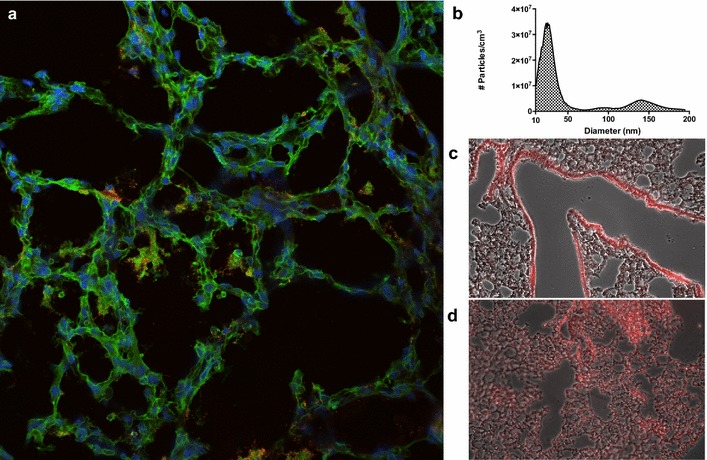
Bacterial and quantum dot deposition. **a** Murine lung section showing intranasal *F. novicida* (*red*) in alveoli. **b** Size distribution of nanoaerosolized quantum dots. **c** Quantum dots delivered intranasally are mainly deposited in the bronchioles and lining mucus. **d** Quantum dot delivered via nanoaerosol penetrate the alveoli and deposit throughout the lung

Previous studies show that *Francisella* targets alveolar type II epithelial cells and macrophages during pulmonary infections so the ability to deliver therapeutics directly to the alveoli would be beneficial [24–26]. Intranasal delivery of *F. novicida* to the lungs was verified to result in localization of bacteria in the alveoli (Fig. 2a). According to MPPD, the small size of nanoaerosols allow for deeper penetration into the lung, specifically to the alveoli. To evaluate this claim, quantum dots were used to trace deposition in the lungs following nanoaerosol exposure and intranasal instillation. Quantum dots are nanocrystals made of semiconducting material ~20 nm in diameter that fluoresce at specified wavelengths and are frequently used as labels [27]. As seen in Fig. 2b, the nanoaerosol generator produced nanoaerosolized particles with a geometric mean diameter of 39 nm, which confirms that the generator is capable of producing particles in the nano-sized range. As predicted, sections of the lungs treated with nanoaerosolized quantum dots show that, when compared to intranasal delivery, particles homogenously penetrate deeper into the lower respiratory tract, including the alveoli and lung parenchyma, instead of mostly being deposited in the bronchioles and mucus (Fig. 2c, d).

**Fig. 3:**
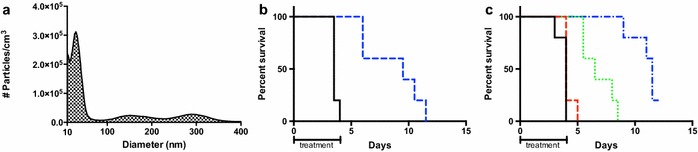
Levofloxacin delivered as nanoaerosol and standard aerosol. **a** Size distribution of nanoaerosolized levofloxacin generated from a 4 mg/mL levofloxacin solution in water. **b** Survival curve of mice treated with nanoaerosolized levofloxacin (*blue*) against 100LD_50_ intranasal *F*. *novicida* infection. **c** Survival curve of mice treated with 3-jet Collison nebulizer aerosolized levofloxacin (30 min—*red*, 1 h—*green*, 2 h—*blue*) against 100LD_50_ intranasal *F. novicida* infection

While quantum dots are a good demonstrative tool, this data is only qualitative in nature and the use of nanoaerosols as therapeutics still requires detailed evaluation. *F. tularensis* is a biothreat agent that is known to be susceptible to treatment with levofloxacin, among other antibiotics [14–17]. Figure 3a shows that the ESN generator is capable of nanoaerosolizing a 4 mg/mL levofloxacin solution in water, producing particles with a geometric mean diameter of 56 nm. The MPPD model predicts a total deposition of 43.5 % for particles of this size: 17.8 % alveolar, 10.3 % tracheobronchial, and 15.4 % nasal (Fig. 1). Since deposition is a physical process that relies on diffusion and impaction, the size of the nanoaerosolized particles will have a similar regional deposition pattern despite the substance or the presence of a complex internal structure being delivered. Since the deposition of the nanoaerosolized quantum dots in the previous experiment supported the MPPD model, a traceable antibiotic was not used. Two additional small peaks with the mode diameters of 150 and 280 nm are present in the size distribution histogram and are most likely the result of particle coagulation.

To determine if the ESN nanoaerosol generator is an effective therapeutic delivery tool, mice were infected with 100LD_50_*F*. *novicida* and subsequently treated with nanoaerosolized aqueous levofloxacin (4 mg/mL) in a conductive whole-body exposure chamber (described in “Methods”) for 4 h a day for 5 days. Despite all mice succumbing to infection, the treated mice showed a significant increase in mean time to death (p < 0.005) compared to the controls: from 3.6 to 8.7 days. The daily total deposited dose, which includes all drug deposited in the respiratory tract regardless of location, was estimated to be approximately 0.42 mg/kg through the use of sample collection PVP filters and the MPPD deposition predictions. For an example calculation from one treatment session, see Additional file 1: Equation S1.

The deposition fractions modeled by the MPPD program were used to estimate deposition here; however, additional characterization of nanoaerosols is necessary to fully understand and accurately calculate the deposition of the particles. Further analysis must account for numerous unknowns, such as the physical properties of the sprayed material within the aerosol and the effect of charge on aerosol deposition, but such experiments are not within the scope of this study.

The Baiera-driven 3-jet Collison nebulizer creates “standard aerosols” composed of particles between 1 and 5 µm in diameter [4]. In this experiment, the time of exposure was altered to change the delivered dose in order to model the nanoaerosol experiment. The standard aerosol of aqueous levofloxacin (4 mg/mL) deposited approximately 0.98 mg/kg/day to the entirety of the respiratory tract during the 2-h treatment group, estimated through the use of an AGI sampler and the MPPD deposition predictions. Of the mice that died in the 2 h per day treatment group, the mean time to death of 10.8 days was significantly longer (p < 0.005) than the 6.8 days mean time to death of the 1 h per day treatment group. The 30 min per day treatment group had a mean time to death of 4.2 days and the control group had a mean time to death of 3.8 days (Fig. 3c). In addition, it was found by histological examination that particles delivered by the ESN nanoaerosol generator do not cause damage to the lungs upon pathologic examination (Additional file 1: Table S1).

For comparison purposes, the lowest effective dose of levofloxacin delivered via intraperitoneal injection and oral administration against 100LD_50_ of *F. novicida* was determined to be 3 and 33 mg/kg, respectively (Additional file 1: Figure S1). A dose of approximately 0.42 mg/kg of nanoaerosolized levofloxacin leads to a mean time to death of 8.7 days compared to the 6.5 days mean time to death of the approximately 0.63 mg/kg dose given via intraperitoneal injection, despite delivering a lower dose. Thus, nanoaerosolized levofloxacin delivered to the lung has more therapeutic value than a higher dose of levofloxacin delivered via intraperitoneal injection.

The 2-h standard aerosol treatment group that received approximately 0.98 mg/kg was not statistically different than the nanoaerosolized treatment group’s, which delivered half the dose at ~0.42 mg/kg (p > 0.05). These results suggest that the use of nanoaerosols significantly decreases the required effective dose of levofloxacin required to rescue mice from a pulmonary *F. novicida* infection by approximately twofold compared to a standard aerosol treatment. Additionally, the amount of solution required to deliver these doses differs significantly. The generation of nanoaerosol in these experiments requires 40 times less volume of sprayed material (~0.5 ml) than what is needed for the generation of standard aerosol (~10 ml). This conservation of material would be particularly useful for otherwise cost prohibitive therapeutics, such as peptides.

Despite these promising results in regard to dose reduction, the therapy is ultimately not viable unless mice can be rescued from a lethal pulmonary *Francisella* infection. Previous reports suggest that liposomes can be used to improve treatments delivered to the respiratory tract and pulmonary delivery of liposome-encapsulated ciprofloxacin enhanced the treatment of *Francisella* infections compared to ciprofloxacin alone [21, 22, 28]. This concept was explored in an attempt to increase survival using nanoaerosol delivery.

**Fig. 4:**
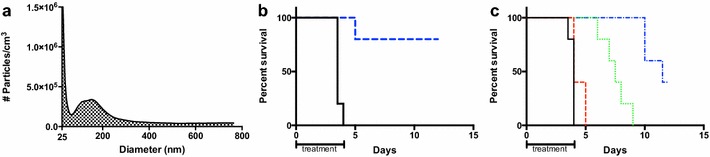
Liposome-encapsulated levofloxacin nanoaerosol and standard aerosol. **a** Size distribution of nanoaerosolized liposome-encapsulated levofloxacin. **b** Survival curve of mice treated with nanoaerosolized liposome-encapsulated levofloxacin (*blue*) against 100LD_50_ intranasal *F*. *novicida* infection. **c** Survival curve of mice treated with aerosolized levofloxacin (30 min—*red*, 1 h—*green*, 2 h—*blue*) against 100LD_50_ intranasal *F. novicida* infection

In order to apply this improvement to the study, liposomes containing levofloxacin were prepared from DPPC, DPPG, and cholesterol precursors (2:1:2 molar ratio) using the well-established thin film dehydration/rehydration technique, including sonication and extrusion, to produce small unilamellar vesicles [29]. As seen in Fig. 4a, the ESN nanoaerosol generator produced nanoaerosolized particles with a geometric mean diameter of 153 nm, which is larger than the nanoaerosolized levofloxacin but still small enough to deliver material to the alveoli. The MPPD model predicts a total deposition of 30.2 % for particles of this size: 8.69 % alveolar, 4.91 % tracheobronchial, and 16.6 % nasal (Fig. 1). The nanoaerosolization of a 4 mg/mL liposome-encapsulated levofloxacin solution increased the percentage of surviving mice to 80 % (4/5 mice rescued) and decreased the estimated delivered dose of levofloxacin to the entire respiratory tract to approximately 0.35 mg/kg per day using the same exposure time of 4 h per day for 5 days (Fig. 4b). The lower delivered dose and decreased percentage delivered to the alveoli compared to the nanoaerosolized levofloxacin suggests that the liposomes themselves may assist with delivery and uptake.

The dose-survival curves were repeated using liposome-encapsulated levofloxacin for the intraperitoneal, oral, and aerosol delivery methods (Fig. 4c; Additional file 1: Figure S1). These curves were not significantly different from those obtained via treatment with naked levofloxacin despite the fact that the 2-h aerosol treatment increased the daily delivered dose from ~0.98 mg/kg per day of levofloxacin to ~1.3 mg/kg per day of liposome-encapsulated levofloxacin. The lowest effective doses for intraperitoneal injection and oral administration of liposome-encapsulated levofloxacin remained about the same as with naked levofloxacin: 3 and 33 mg/kg per day, respectively. These findings are inconsistent with the literature, which asserts that liposomes enhance intraperitoneal and oral treatments; however, perhaps delivering the minimal, barely- effective doses of liposome-encapsulated levofloxacin does not make a significant difference when administering this particular treatment by intraperitoneal or oral routes against pulmonary *Francisella* infections.

The lack of a significant change in the standard aerosol dose survival curve between unencapsulated and liposome-encapsulated levofloxacin seemed to contradict the results reported by Wong et al. [21, 30]. To begin to address this, further investigation showed that over 50 % of the liposomes had burst and a significant portion of the levofloxacin had reverted back to the unencapsulated form as a result of the 3-jet Collison nebulizer aerosolization process. This is consistent with previous reports as to the membrane disruption caused by this type of nebulizer [30, 31]. Since the Collison nebulizer aerosolization process resulted in such a large reduction in the concentration of intact liposomes, the nanoaerosolized liposomal levofloxacin dose cannot be accurately compared to the dose of liposomes aerosolized by this particular aerosolization process. However, this finding supports the notion that the ESN nanoaerosol generator is gentler on sprayed material than a Collison jet nebulizer, as seen by the preservation of liposomes, and would be useful in delivering fragile biological substances. Finally, it can be concluded from the survival curves that ~0.35 mg/kg of nanoaerosolized liposomal levofloxacin is statistically equivalent in terms of survival to 3 mg/kg liposomal levofloxacin delivered via intraperitoneal injection and 33 mg/kg liposomal levofloxacin by oral delivery (p > 0.005). This data is equivalent to an 8-fold reduction in the minimum effective treatment dose compared to the intraperitoneal delivery method and 94-fold reduction compared to the oral delivery method.

## Conclusion

It has previously been established that aerosolized therapeutics can improve upon the traditional delivery methods of oral and intraperitoneal injection. However, this study demonstrates that nanoaerosols (less than 200 nm) are potentially the next phase of improvement in drug delivery.

One significant benefit of nanoaerosolized therapeutic agents is the very small total volume required for each treatment. Through the method of ESN aerosol generation alone, nanoaerosols require 40 times less initial volume to spray than traditional nebulizers. This could be a significant boon for treatments in which the required therapeutics are extremely expensive, such as peptides or biologics. Due to the difficulty in delivering high doses of therapeutics via nanoaerosols because of the small amount of drug mass contained in the nanometer-sized particles, liposomes are a useful tool in enhancing deposition and uptake of the delivered therapeutic in the lungs. Nanoaerosol generation is gentler on sprayed therapeutic compounds than the Collison jet nebulizer, as seen by the preservation of liposomes, and therefore is compatible with the use of liposome-encapsulated therapeutics or other fragile materials.

Nanoaerosols have proven to reduce the required effective dose of levofloxacin to rescue mice from a pulmonary *F*. *novicida* infection. Nanoaerosolized liposome- encapsulated levofloxacin results in an 8-fold reduction compared to the intraperitoneal delivery method and 94-fold reduction compared to the oral delivery method. Nanoaerosolized levofloxacin is also as effective as twice the dose of levofloxacin aerosolized via a 3-jet Collison nebulizer. This result is most likely due to the direct delivery some fraction of the antibiotic to the lower respiratory tract and the alveolar space, which is the initial site of *Francisella* infection in this model. These results illustrate the significant benefit of direct delivery to the site of infection in the alveoli. In addition, the delivery of nanoaerosols to the lung showed no evidence of causing tissue damage in mice. These results are highly encouraging to pursue the further development of nanoaerosol-based therapeutic delivery, especially for its ability to achieve a therapeutic resolution of infection with a significantly reduced dose and the small net amount of therapeutic used. This technology could assist patients suffering from pneumonia, cystic fibrosis, and potentially other systemic diseases, such as diabetes, by enabling pulmonary delivery of medication at a significantly reduced dose, which could lead to a reduction in cost and the number or severity of side effects. Future studies could explore the range of therapeutics that can be delivered via the nanoaerosol generator and other applications to which the technology can be applied. More detailed studies of the biophysical characteristics of the particles and their deposition could also be performed. In addition, the pharmacokinetics and pharmacodynamics of delivered nanoaerosolized therapeutics should be further investigated in order to fully develop the benefits demonstrated in this study.

## Methods

### Deposition modeling

Respiratory deposition probabilities for aerosolized particles in BALB/c mice were calculated using the Multiple Path Particle Dosimetry (MPPD) model, version 3.0 (Chemical Industry Institute of Toxicology, Research Triangle Park, NC). MPPD 3.0 software models the deposition of various sized aerosolized particles within a mouse respiratory tract based on extensive data from previously published studies [23, 32]. The parameters of mouse model, MMAD and a size range from 0.01 to 1 μm were used. Estimated respiratory values of BALB/c mice determined by Flandre et al. were entered into the program for modeling purposes (Additional file 1: Equation S1) [33].

### Nanoaerosol generation

The ESN nanoaerosol generator was used in this study as previously described [3, 5]. Briefly, a sample suspended or dissolved in water with a conductivity of less than 200 μS/cm was sprayed at a positive potential while ethanol was sprayed at a negative potential. Conductivity was measured with a conductivity meter (Oakton Con 11 Series, Thermo Scientific, Waltham, MA) possessing a modified probe to allow for the measurement of low volume samples. To accelerate atomization, the positively charged capillary had a pressure of between 7 and 9 cm H_2_O and a current of 95 nA applied. The pressure and current in the negatively charged capillary were 3.6 cm H_2_O and 40 nA, respectively. The volumetric yield of nanoaerosol was 2 L per minute. Mice were exposed to nanoaerosols for 4 h in a conductive whole body exposure unit (described below) attached to the generator output by conductive tubing.

### Whole body exposure unit

This specialized chamber was developed at GMU to enable the delivery of nanoaerosols to five mice at a time. The chamber was produced by taking a 1 L acrylic, latched induction chamber (Vetequip, Inc, 941443), with the dimensions 3.75″ × 4.5″ × 3.75″, and drilling out the plastic inlet and outlet ports to be replaced with brass fittings as shown in Additional file 1: Figure S2. Then, taping off a “window” on each side to allow for observation of the mice, the inside of the box was coated with Total Ground Carbon Conductive Coating (MG Chemicals, 838–340 g), including the floor and lid, to reduce the deposition of nanoaerosol particles. The outside of the box was coated with Super Shield Silver Coated Copper Conductive Coating (MG Chemicals, 843–140 g). Using a piece of copper tape, the lid was connected to the base of the box to ensure connection (not shown). This box can comfortably hold five mice for whole body exposure to nanoaerosols, which are delivered to the box via conductive tubing to the inlet port. Post chamber sampling can be done from the outlet port.

### Aerosol sizing

Nanoaerosol particles were sized using a Scanning Mobility Particle Sizer (SMPS, TSI Incorporated, Shoreview, MN) to measure air particle size distribution in the range of 20–1000 nm. The SMPS is composed of an Ultrafine Water-based Condensation Particle Counter (model 3786), an Electrostatic Classifier (model 3080), a Long Differential Mobility Analyzer (model 3081), and the Aerosol Instrument Manager^®^ software (TSI Incorporated, Shoreview, MN).

### Quantum dots deposition

Six to eight week old female BALB/c mice (Harlan, Frederick, MD) were given a 46 nM quantum dot (20 nm Qdot 705, Life Technologies, Grand Island, NY) solution by either a 4-h nanoaerosol spray or a single 40 μL intranasal dose. Controls received 40 μL of PBS intranasally. Mice rested for 2 h before being administered a ketamine–xylazine cocktail. While under anesthesia, mice were euthanized and lungs were perfused in situ with 10 mL of PBS followed by 20 mL of 4 % depolymerized paraformaldehyde. Lungs were harvested and underwent cryosectioning (10 μm thick sections with Thermo Scientific HM550 cryostat, Waltham, MA) and imaged using a confocal microscope (Nikon Eclipse TE2000-U, Melville, NY).

### Bacterial strains

*Francisella novicida* (ATCC 15482) was obtained from American Type Culture Collection (Manassas, VA). All bacteria were streaked onto tryptic soy agar with 0.1 % cysteine (TSAC) or Chocolate Agar (GC Agar II with Isovitalex, BD Biosciences, Franklin Lakes, NJ) plates and single colonies were inoculated into tryptic soy broth with 0.1 % cysteine (TSBC) or Brain Heart Infusion, pH 6.8 (BHI) broth (TekNova, Hollister, CA). Cultures were incubated at 37 **°**C overnight with liquid cultures at 250 rpm.

### *Francisella* in alveoli

Six to eight week old female BALB/c mice (Harlan, Frederick, MD) were infected with *F. novicida* intranasally. Following euthanasia, lung sections were stained with DAPI to observe the nuclei, FITC phalloidin (green) to observe the cellular actin, and goat anti-*Francisella tularensis* affinity purified polyclonal antiserum (DD-33, AB-AG-FTUL, Department of Defense Critical Reagents Program) was used as the primary antibody. The primary antibody was detected using donkey anti-goat IgG (H + L) secondary antibody, Alexa Fluor 594 (red) conjugate (Life Technologies, Fredrick, MD). The blue, green and red images were merged together to produce a composite image.

### Murine infection

Six to eight week old female BALB/c mice (Harlan, Frederick, MD) were infected intranasally with 50 μL of PBS containing 100LD_50_ of *F. novicida* (approximately 1000 CFU) following IACUC approved protocols. Bacterial inoculum concentrations were verified retrospectively via plating on chocolate agar. Mice were monitored twice daily for the duration of the study and assigned health scores according to institutional standards. Human-equivalent  endpoints were used at a designated health score cut off which corresponds to significant weight loss, dehydration, lethargy, and lack of responsiveness. Mice were euthanized via CO_2_ asphyxiation followed by cervical dislocation. Occasional mice declined rapidly between health checks and succumbed to infection prior to euthanasia.

### Antibiotic treatments

Three hours post infection treatment was initiated and continued once a day for 5 days by (i) a 100 µL intraperitoneal injection, (ii) a 100 µL orally administered dose, (iii) a 0.5–2 h aerosol treatment in a whole body exposure chamber (utilizing approximately 20 mL to create aerosol), or (iv) a 4 h nanoaerosol treatment in a whole body exposure (utilizing approximately 500 µL to create nanoaerosol). Aerosol treatment was administered via a three-jet Collison nebulizer provided as part of the Biaera whole body exposure system (Biaera Technologies, Hagerstown, MD). Nanoaerosol treatment was administered as described above. Dosages were based on the average weight of all mice in the experimental run. Estimated respiratory values of mice determined by Flandre et al. were used in conjuction with MPPD to calculate approximate deposition in lungs (Additional file 1: Equation S1) [33].

### Aerosol sampling

Aerosol samples from the Biaera aerosol generator were collected in distilled water contained within an all-glass impinger (AGI). Nanoaerosol samples from the ESN nanoaerosol generator were collected on polyvinylpyrrolidone (PVP) filters that were dissolved in 100 µL of distilled water for measurements. PVP filters provide high capturing efficiency and are ideal for sample analysis because they are chemically inert [34, 35]. Collected levofloxacin samples from both methods were diluted with distilled water and the antibiotic concentration was measured at a wavelength of 288 nm on a NanoDrop spectrophotometer (Thermo Scientific, Waltham, MA) against a standard curve. Liposome-encapsulated levofloxacin samples were diluted in ethanol as opposed to distilled water to disrupt the liposome membranes prior to measurements and were measured at 300 nm.

### Liposome preparation

1,2-dipalmitoyl-sn-glycero-3-phosphocholine (DPPC) and 1,2-dipalmitoyl-sn-glycero-3-phosphoglycerol (DPPG) was acquired from Echelon Biosciences Incorporated (Salt Lake City, UT). Cholesterol was acquired from Sigma-Aldrich (St. Louis, MO). Liposomes containing a saturated solution of levofloxacin in water were prepared from DPPC, DPPG, and cholesterol precursors (2:1:2 molar ratio) using the well-established thin film dehydration/rehydration technique followed by sonication and extrusion to produce small unilamellar vesicles [29, 36]. Liposomes were sized using a qNano particle analyzer (iZon, Oxford, United Kingdom) and shown to have a mean and mode diameter of approximately 172.1 and 124 nm, respectively (Additional file 1: Figure S3). Levofloxacin concentration within liposomes was measured using a NanoDrop spectrophotometer (Thermo Scientific, Waltham, MA) as described above. Liposomes were burst with ethanol to determine final levofloxacin concentration and diluted to a concentration of 4 mg/mL for administration.

### Histopathologic examination

Lungs, livers, and spleens were harvested from four different mice: (1) an uninfected, untreated naïve mouse; (2) an uninfected, naïve mouse treated with liposome-encapsulated water; (3) an infected mouse treated with liposome- encapsulated levofloxacin; and (4) an infected, untreated mouse. Formalin fixed organs were submitted to Experimental Pathology Laboratories, Inc. (Sterling, VA) for processing, hematoxylin and eosin staining, and histopathologic evaluation. Samples were randomly assigned numbers to ensure blind scoring by the pathologist.

### Ethics statement

All animal experiments included in this manuscript were approved by and conducted in compliance with regulations of the Institutional Animal Care and Use Committee (protocol #0253) of George Mason University. All experiments were carried out in accordance with the National Research Council’s Guide for the Care and Use of Laboratory Animals (2011) and the Public Health Service Policy on Humane Care and Use of Laboratory Animals (2002).

### Statistical analysis

The survival curves were analyzed using the Mantel-Cox test, which is used to test the null hypothesis that survival curves are not different between groups. Low p values correspond with a rejection of the null hypothesis, which means that there is a statistical difference between the survival data of different treatment groups. This test does not assume a normal distribution, allows for censored data, and is based off of the Chi squared test, which allows for a minimum of five samples. Each experimental group contained five mice and experiments were repeated to confirm results.

## Additional file

10.1186/s12951-016-0182-0 **Equation S1.** Example Dosing Calculation. This calculation shows the equation for estimating the nanoaerosolized levofloxacin dose deposited in each mouse during one spray session and the general principle behind all of the dosing calculations in this study. The spray variable was determined through the use of PVP filters for nanoaerosol treatments and AGI for standard aerosol treatments and varies for each treatment. The ITV (inspiratory tidal volume) and RR (respiratory rate) variables use mouse physiological data measured by Flandre et al. [25] The mass variable is the average weight of all mice in that particular run of the experiment. The deposition variable is the total respiratory tract deposition percentage as determined by MPPD based on the mean particle diameter, in this case 56 nm. **Figure S1.** Levofloxacin and Liposome-Encapsulated Levofloxacin Survival Curves from Traditional Delivery Methods. The survival curves of intraperitoneal and oral levofloxacin (A, B) and liposome-encapsulated levofloxacin (C, D) against 100LD_50_ intranasal Francisella tularensis subsp. novicida shows the lowest effective dose is approximately 3 and 33 mg/kg, respectively. **Table S1.** Histopathologic Scoring of Murine Lungs, Livers, and Spleens. Histopathologic findings of pathologist are shown. A corresponds with an infected, untreated mouse, B corresponds with an infected mouse treated with nanoaerosolized liposome-encapsulated levofloxacin, C corresponds with an uninfected mouse treated with nanoaerosolized liposome-encapsulated water, and D corresponds with an uninfected, untreated mouse. The magnitude of inflammatory or degenerative lesions was graded on a scale of 1 to 5, with Grade 1 being minimal and Grade 5 being severe. P is indicative of bacteria being present. **Figure S2.** Conductive Whole Body Exposure Chamber. Modified whole body exposure unit that allows for greater conductivity and less loss of nanoaerosol during treatments. (1) Aerosol input/output ports, (2) viewing window, (3) conductive paint, and (4) latch. **Figure S3.** qNano Liposome Analysis. qNano analysis of liposome-encapsulated levofloxacin prior to nanoaerosol generation shows a mean and mode diameter of 172.1 and 124.9 nm, respectively.
