# Supplementary material for: Nanoaerosols reduce required effective dose of liposomal levofloxacin against pulmonary murine Francisella tularensis subsp. novicida infection
Source: J Nanobiotechnology. 2016 Apr 18;14:29. doi: 10.1186/s12951-016-0182-0 (PMC4835885; doi:10.1186/s12951-016-0182-0)
Supplement: Supplementary file 1 — 10.1186/s12951-016-0182-0 Equation S1. Example Dosing Calculation. This calculation shows the equation for estimating the nanoaerosolized levofloxacin dose deposited in each mouse during one spray session and the general principle behind all of the dosing calculations in this study. The spray variable was determined through the use of PVP filters for nanoaerosol treatments and AGI for standard aerosol treatments and varies for each treatment. The ITV (inspiratory tidal volume) and RR (respiratory rate) variables use mouse physiological data measured by Flandre et al. [25] The mass variable is the average weight of all mice in that particular run of the experiment. The deposition variable is the total respiratory tract deposition percentage as determined by MPPD based on the mean particle diameter, in this case 56 nm. Figure S1. Levofloxacin and Liposome-Encapsulated Levofloxacin Survival Curves from Traditional Delivery Methods. The survival curves of intraperitoneal and oral levofloxacin (A, B) and liposome-encapsulated levofloxacin (C, D) against 100LD50 intranasal Francisella tularensis subsp. novicida shows the lowest effective dose is approximately 3 and 33 mg/kg, respectively. Table S1. Histopathologic Scoring of Murine Lungs, Livers, and Spleens. Histopathologic findings of pathologist are shown. A corresponds with an infected, untreated mouse, B corresponds with an infected mouse treated with nanoaerosolized liposome-encapsulated levofloxacin, C corresponds with an uninfected mouse treated with nanoaerosolized liposome-encapsulated water, and D corresponds with an uninfected, untreated mouse. The magnitude of inflammatory or degenerative lesions was graded on a scale of 1 to 5, with Grade 1 being minimal and Grade 5 being severe. P is indicative of bacteria being present. Figure S2. Conductive Whole Body Exposure Chamber. Modified whole body exposure unit that allows for greater conductivity and less loss of nanoaerosol during treatments. (1) Aerosol input/o [file 12951_2016_182_MOESM1_ESM.pdf]

Knowns

$Spray - 146\ {}^{\mu g}/_{hr}$
$Flow\ Rate - 108\ {}^{L}/_{hr}$
$ITV - 0.219\ {}^{mL}/_{breath}$
$RR - 273.1\ {}^{breath}/_{min}$
$Mass - 20\ g$
$Total\ Deposition - 43.5\%\ or\ 0.435$

$$0.219\ {}^{mL}/_{breath} * 273.1\ {}^{breath}/_{min} = 59.81\ {}^{mL}/_{min}\ or\ 3.588\ {}^{L}/_{hr}$$

$$\frac{146\ {}^{\mu g}/_{hr}}{108\ {}^{L}/_{hr}} = 1.35\ {}^{\mu g}/_{L} * 3.588\ {}^{L}/_{hr} = 4.8438\ {}^{\mu g}/_{hr}$$

$$0.435 * 4.8438\ {}^{\mu g}/_{hr} = 2.107\ {}^{\mu g}/_{hr} * 4\ hr = 8.428\ {}^{\mu g}/_{day}\ \left(\sim 0.42\ {}^{mg}/_{kg}\right)$$

Equation S1. Example Dosing Calculation. This calculation shows the equation for estimating the nanoaerosolized levofloxacin dose deposited in each mouse during one spray session and the general principle behind all of the dosing calculations in this study. The spray variable was determined through the use of PVP filters for nanoaerosol treatments and AGI for standard aerosol treatments and varies for each treatment. The ITV (inspiratory tidal volume) and RR (respiratory rate) variables use mouse physiological data measured by Flandre *et al.* [25] The mass variable is the average weight of all mice in that particular run of the experiment. The deposition variable is the total respiratory tract deposition percentage as determined by MPPD based on the mean particle diameter, in this case 56 nm.

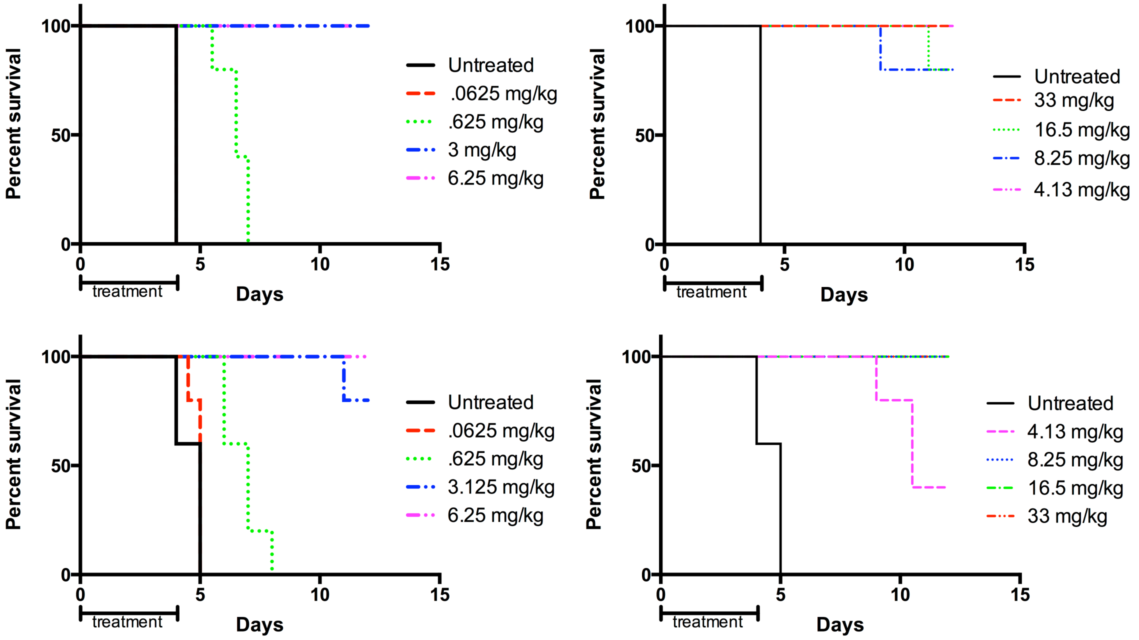

Figure S1. Levofloxacin and Liposome-Encapsulated Levofloxacin Survival Curves from Traditional Delivery Methods. The survival curves of intraperitoneal and oral levofloxacin (A, B) and liposome-encapsulated levofloxacin (C, D) against 100LD$_{50}$ intranasal *Francisella tularensis* subsp. *novicida* shows the lowest effective dose is approximately 3 and 33 mg/kg, respectively.

Mouse ID	Findings	Grade
A	**LIVER**	
	Necrosis, random, with subacute inflammation	3
	Intracellular bacteria	P
	**SPLEEN**	
	Necrosis, with subacute inflammation	4
	**LUNG**	
	Necrosis, with subacute inflammation	3
	Intracellular bacteria	P
	Hemorrhage and edema	3
	Foreign material, pigmented	3
B	**LIVER**	
	Necrosis, random, with subacute inflammation	3
	**SPLEEN**	
	Inflammation, subacute	4
	**LUNG**	
	Inflammation, subacute, perivascular	4
	Hemorrhage and edema	3
C	**LIVER**	
	Not remarkable	-
	**SPLEEN**	
	Not remarkable	-
	**LUNG**	
	Not remarkable	-
D	**LIVER**	
	Infiltrate, mononuclear cell	1
	**SPLEEN**	
	Not remarkable	-
	**LUNG**	
	Not remarkable	-

Table S1. Histopathologic Scoring of Murine Lungs, Livers, and Spleens. Histopathologic findings of pathologist are shown. A corresponds with an infected, untreated mouse, B corresponds with an infected mouse treated with nanoaerosolized liposome-encapsulated levofloxacin, C corresponds with an uninfected mouse treated with nanoaerosolized liposome-encapsulated water, and D corresponds with an uninfected, untreated mouse. The magnitude of inflammatory or degenerative lesions was graded on a scale of 1 to 5, with Grade 1 being minimal and Grade 5 being severe. P is indicative of bacteria being present.

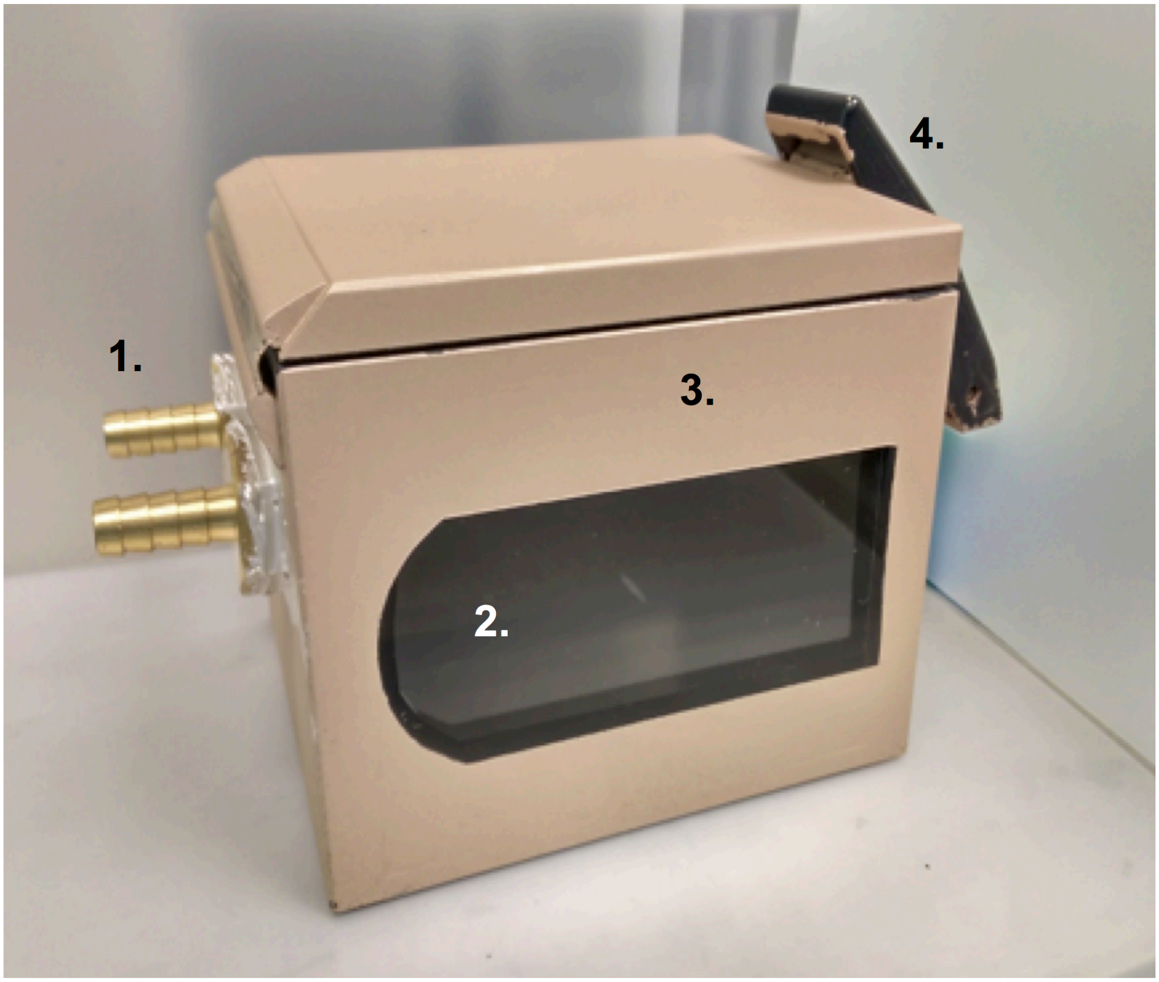

Figure S2. Conductive Whole Body Exposure Chamber. Modified whole body exposure unit that allows for greater conductivity and less loss of nanoaerosol during treatments. 1) Aerosol input/output ports, 2) viewing window, 3) conductive paint, and 4) latch.

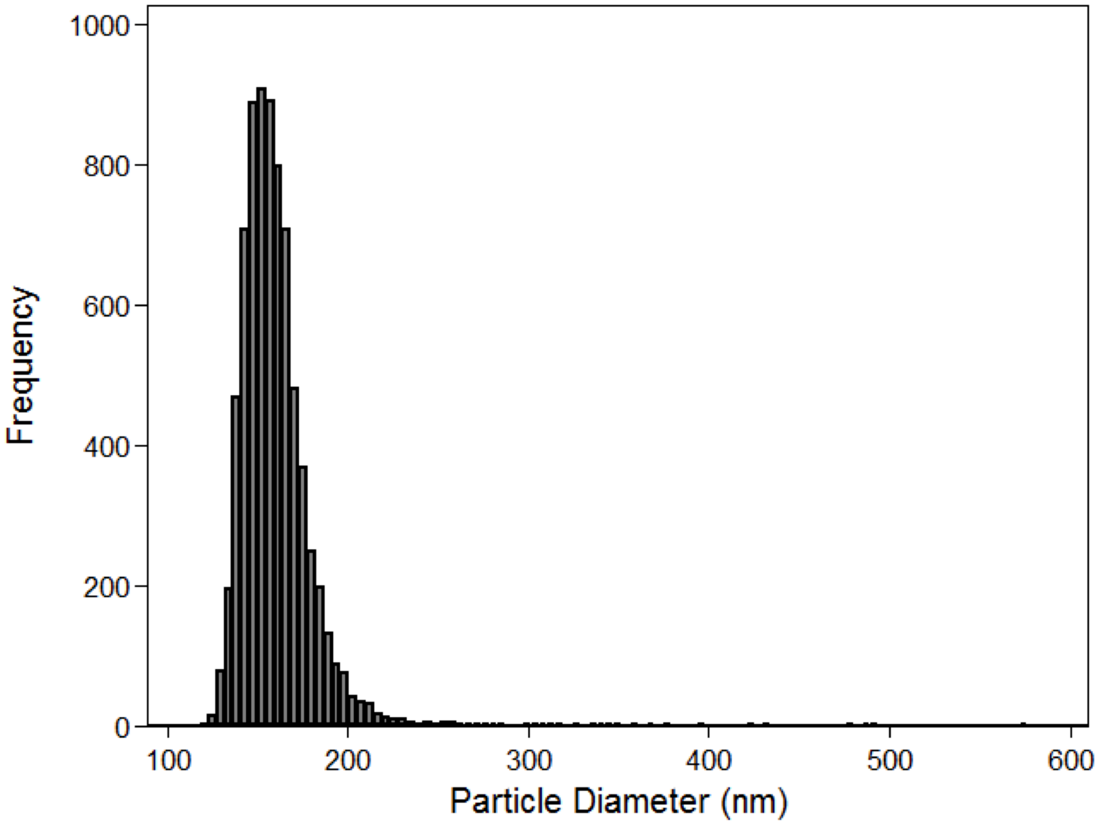

Figure S3. qNano Liposome Analysis. qNano analysis of liposome-encapsulated levofloxacin prior to nanoaerosol generation shows a mean and mode diameter of 172.1 nm and 124.9 nm, respectively.
